# Improved polarized light microscopic detection of gouty crystals via dissolution with formalin and ethylenediamine tetraacetic acid

**DOI:** 10.1038/s41598-023-34570-5

**Published:** 2023-05-09

**Authors:** Ruedee Hemstapat, Peeradon Duangiad, Borwornporn Tangketsarawan, Thitiya Phuagpan, Sinthida Chienwiwattanawong, Nuttinee Tangsrianugul, Akio Ojida, Jirarut Wongkongkatep

**Affiliations:** 1 Department of Pharmacology, Faculty of Science, Mahidol University, 272 Rama 6 Road, Bangkok, 10400 Thailand; 2 Department of Biotechnology, Faculty of Science, Mahidol University, 272 Rama 6 Road, Bangkok, 10400 Thailand; 3 Graduate School of Pharmaceutical Sciences, Kyushu University, 3-1-1 Maidashi, Higashi-Ku, Fukuoka, 812-8582 Japan

**Keywords:** Rheumatology, Chemistry

## Abstract

Conventional polarized light microscopy has been widely used to detect gouty crystals, but its limited sensitivity increases the risk of misidentification. In this study, a number of methods were investigated to improve the sensitivity of polarized light microscopy for the detection of monosodium urate monohydrate (MSUM) and calcium pyrophosphate dihydrate (CPPD) crystals. We found that coating glass slides with poly-*L*-lysine, a positively charged polymer, improved the attachment of crystals to the glass surface, resulting in clearer crystal images compared to non-coated slides. Additionally, the sensitivity of detection was further enhanced by selective dissolution, in which 40% v/v formalin phosphate buffer was employed to dissolve MSUM crystals but not CPPD while 10% ethylenediamine tetraacetic acid (EDTA) was employed to dissolved CPPD but not MSUM. The other possible interferences were dissolved in both EDTA and formalin solution. These methods were successfully applied to detect gouty crystals in biological milieu, including spiked porcine synovial fluid and inflamed rat subcutaneous air pouch tissues.

## Introduction

Gout is a chronic form of arthritis caused by monosodium urate monohydrate (MSUM) deposition in the joint cavity^1^. This condition results in painful flares that may exacerbate over time. Approximately 15% of gout patients develop tophi, subcutaneous nodules composed of MSUM crystals that can cause unremitting joint inflammation and potential joint erosion and deformity^2^. Another form of crystal-induced arthritis is pseudogout, also known as calcium pyrophosphate dihydrate (CPPD) deposition disease which induce similar clinical presentation as gout^3^. CPPD crystals consist of two forms which are triclinic CPPD or t-CPPD and monoclinic CPPD or m-CPPD. Both forms of CPPDs were found in the affected joint of patients^4^. Differential birefringence by polarized light microscope is the standard method for synovial fluid analysis in the search for MSUM and CPPD crystals. Although MSUM crystals are strongly birefringent, CPPD crystals are poorly birefringent^5,6^. There are some reports mentioned that a relatively higher birefringence was found due to co-aggregation or contamination of other crystals^7,8^. Therefore, it would be problematic to determine type of crystal present in synovial fluid examination. This conventional method is also limited by high false negative rates (2.8% for gout and 7.9% for pseudogout patients)^9^ depending on the experience in crystal analysis and the level of training of the observers^10^. This problem was initially indicated back in 1986 by Schumacher et al.^11^ and several other studies proposed for quality control of synovial fluid analyses^12,13^. Thus far, various strategies have been developed to improve the sensitivity of MSUM and CPPD detection, including the use of Fv antibodies^14^, fluorescent probes^15^, nanostructured TiO_2_ material^16^, X-ray dark field radiography^17^, optical diffraction tomography^18^, lens-free polarized microscopy^19^ and dual energy computed tomography^20^. These methods provide high sensitivity for gouty crystal detection, but they require specific reagents and sophisticated instruments.

Studies have reported that the use of certain tissue processing techniques can lead to the disappearance of MSUM and CPPD crystals. For instance, formalin fixation has been found to result in losses of MSUM crystals^21^, while preservation of synovial fluid with EDTA has been shown to decrease the identification rate of CPPD crystals by 66.7%^22^. However, while these solutions may not be ideal for the preservation of gouty crystals, we surmised that they could be exploited to enhance the sensitivity and specificity of MSUM detection using microscopy. For instance, to confirm that the crystals are truly MSUM, the sample may be treated with formalin and observed using polarized light microscopy—the disappearance of the crystals would indicate that the identification is correct. Crystals that survive the formalin treatment but disappear in the presence of EDTA are likely to be CPPD. Therefore, in this study, we systematically investigated the ability of formalin and EDTA solutions to dissolve MSUM and CPPD crystals, and evaluated whether this selective dissolution strategy could promote accurate identification of gouty crystals, especially those in biological milieu such as synovial fluid.

Continuous observation of microscopic crystals in low-viscosity media, such as water, presents a challenge due to increased Brownian motion^23^. Exacerbating this problem is the strong negative charge of gouty crystals^24^, which prevents their attachment to the negatively charged surface of glass slides^25^. A potential strategy is to coat the glass surface with cationic polymers, such as poly-*L*-lysine (PLL), which is a homopolymer of *L*-lysine and exhibits positive charge across a wide pH range. This polymer has demonstrated strong adherence to negatively charged surfaces in water, and therefore has been widely used to immobilize cells and proteins for microscopic observation^26^. Another promising candidate of coating material is chitosan, which is strongly positively charged, low cost, and eco-friendly^27^. Coating slides with the aforementioned cationic polymers, therefore, would limit the movement of crystals in media, thereby improving the clarity of images obtained with polarized light microscopy.

## Results and discussion

### Gouty crystals characterization and dissolution by weight analysis

**Figure 1:**
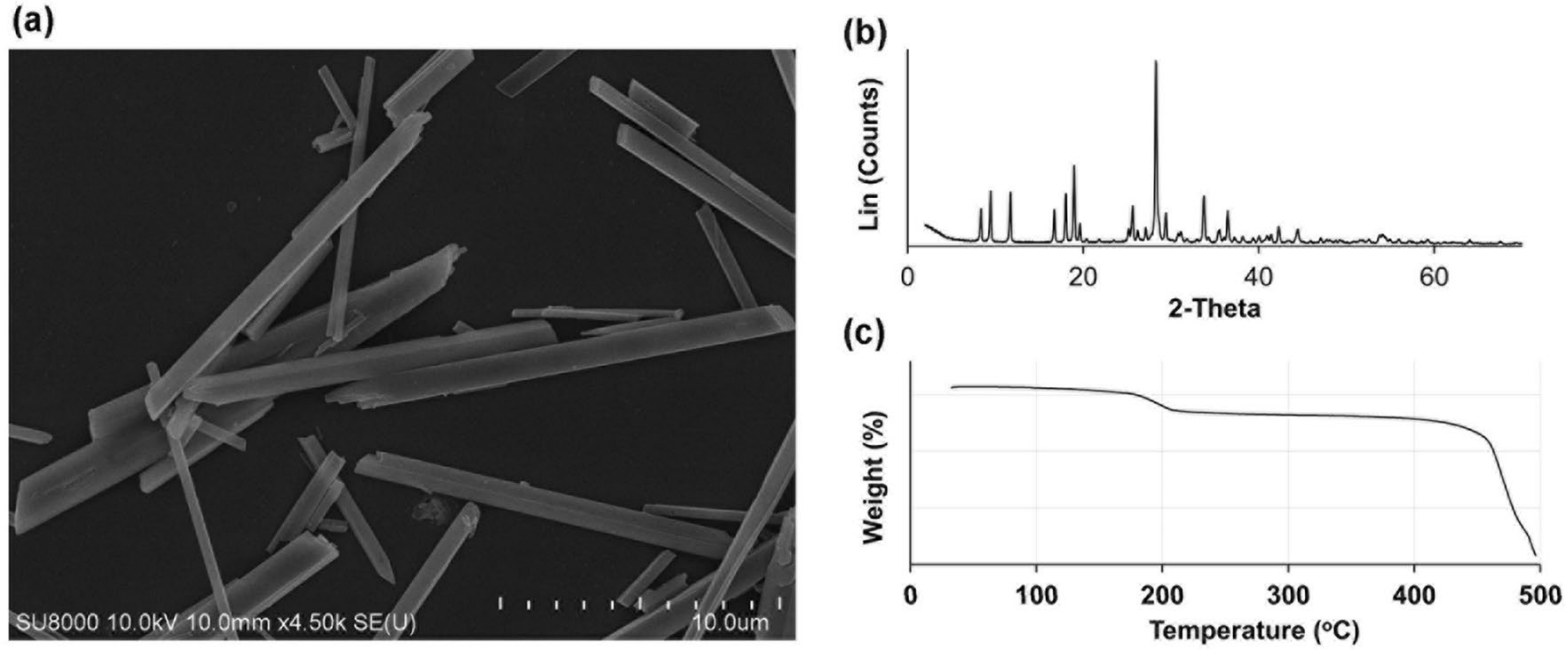
Crystal characteristics. (**a**) Scanning electron micrograph, (**b**) powder X-ray diffraction pattern and (**c**) thermal gravimetric analysis profile of the synthesized MSUM crystals.

The synthesized MSUM crystals displayed a needle-like shape but lacked needle points, which might have been fragmented during filtration. Scanning electron microscopy (SEM) revealed that the crystals possessed smooth surfaces (Fig. 1a). The powder X-ray diffraction (XRD), FTIR, and Raman spectra acquired were consistent with the literature, confirming that the crystals were pure MSUM^28^ (Fig. 1b, see Supplementary Fig. S1 online). According to thermal gravimetric analysis (TGA), the weight of the crystals decreased by 8.7% upon raising the temperature from 108 to 252 °C. This value was nearly identical to the theoretical weight reduction due to the loss of one water molecule per MSUM, indicating that the crystals were monohydrate (Fig. 1c). The crystals of CPPDs were prepared as previously described^15^ (see Supplementary Page S2 online).

**Figure 2:**
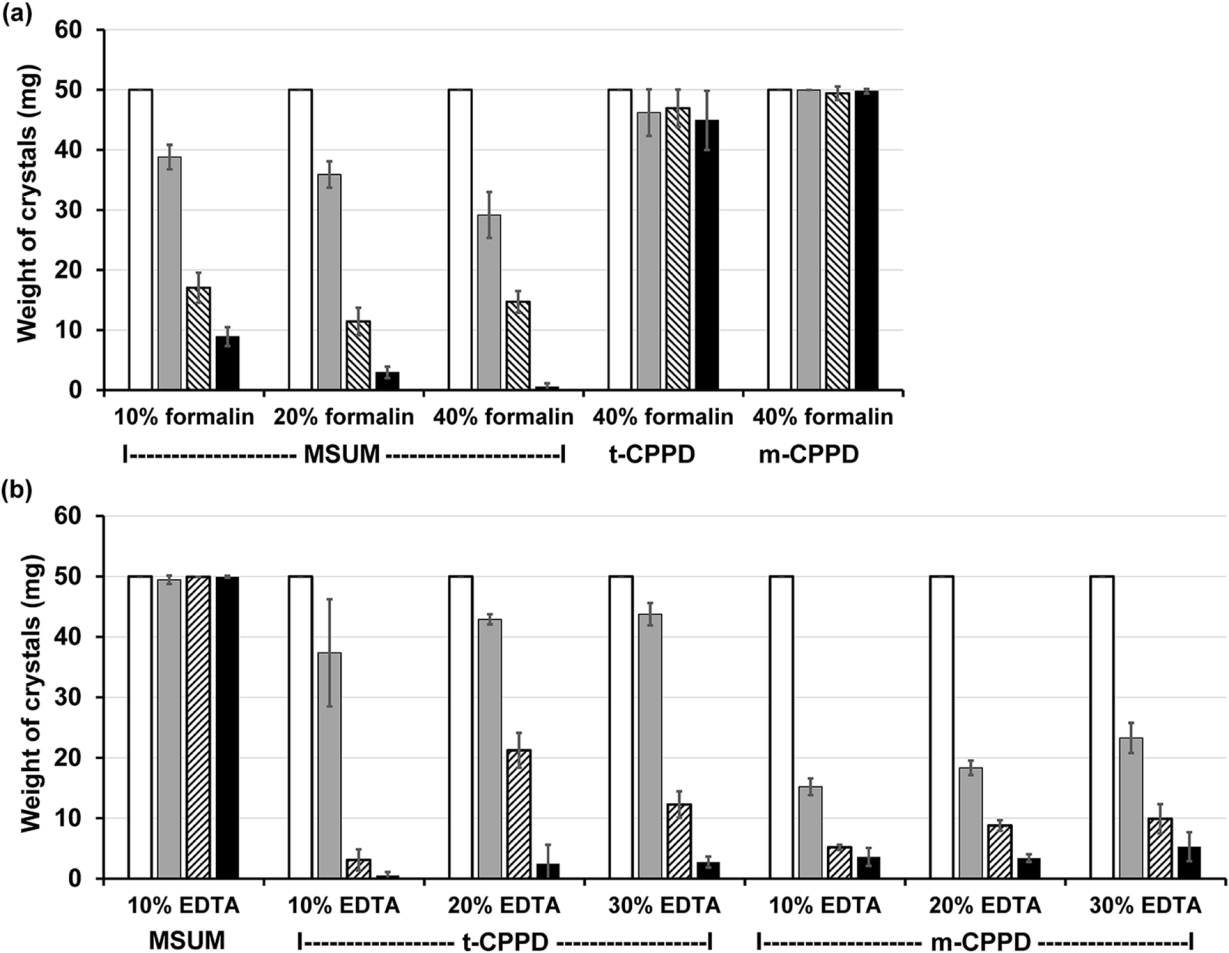
Analysis of crystal weights in the presence of formalin or EDTA. Weight loss of MSUM, t-CPPD and m-CPPD crystals before (white) and after shaking with (**a**) 10–40% v/v formalin phosphate buffer (10 mM, pH 7.4) or (**b**) 10–30% EDTA aqueous solution (pH 9.0) at 25 °C, 200 rpm for 0 (gray), 15 (hatched) and 30 min (black). The data represent average values and error bars represent SD (n = 3).

It had been suspected that MSUM and CPPD crystals dissolved during formalin fixation and decalcification of tissues, respectively^21,22^. Herein, we systematically evaluated the solubility of MSUM and CPPD crystals in formalin phosphate buffer and EDTA solution, which are typically used in the tissue processing^29^. First, using gravimetric analysis, we measured the weight loss of MSUM crystals upon being shaken with different concentrations of formalin in 10 mM phosphate buffer (pH 7.4) (Fig. 2a). The results show that the extent of weight reduction increased as formalin concentration increased. Notably, the solid weight dropped from 50 to 21 mg immediately upon the addition of 40% v/v formalin, suggesting a rapid and high solubility of MSUM in 40% v/v formalin solution. The solid weight reduced to less than 1 mg after 30 min of shaking (Fig. 2a), implicating formalin concentration as an important factor for the dissolution of MSUM crystals.

However, soaking MSUM crystals in a 10% w/v EDTA solution (pH 9.0) did not significantly reduce the weight, consistent with the reported poor solubility of MSUM crystals in aqueous solutions with pH lower than 10.3. MSUM will dissolved rapidly when pH is higher than 10.3 due to the second deproponation according to the second p*K*_a_ of the uric acid^30,31^ (Fig. 2b). On the other hand, the solubility of t-CPPD and m-CPPD crystals was found to be negligible in 40% v/v formalin phosphate buffer, while it was high in the 10% w/v EDTA solution (pH 9.0), possibly due to ability of EDTA to form a strong complex with calcium ion. In addition, unlike the solubility of MSUM in formalin solution, the concentration of EDTA had no effect on the dissolution rate of CPPDs crystals (Fig. 2b). XRD analysis revealed almost pure MSUM when mixed crystals of MSUM and CPPDs were treated with 10% w/v EDTA solution (pH 9.0) for 30 min. On the other hand, the mixed crystals showed almost pure CPPDs after contact with 40% v/v formalin phosphate buffer (10 mM, pH 7.4) for 30 min (see Supplementary Fig. S2 online). Collectively, these results indicate that soaking MSUM crystals in 10% w/v EDTA solution (pH 9.0) could be an effective strategy for eliminating t-CPPD and m-CPPD, and enhance the sensitivity of MSUM detection via polarized light microscopy.

### Immobilization of gouty crystals on PLL-coated glass slides

**Figure 3:**
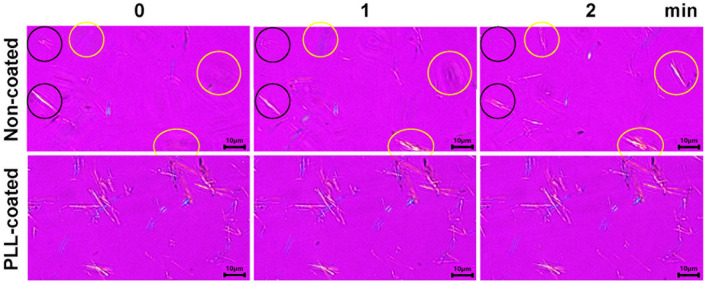
Images of comparing the appearance of MSUM crystals on PLL-coated and non-coated glass slides. Polarized light microscopic images of MSUM on a non-coated glass slide (above) and a 0.01% w/v PLL-coated glass slide (below). Scale bars represent 10 μm. Yellow circles indicate the crystals that gradually appeared during the imaging. Black circles indicate the crystals that faded out during the observation. Differential interference contrast (DIC) images are available in Supplementary Fig. S3 online.

In this study, we proposed selective dissolution of gouty crystals as a strategy to improve the accuracy of their detection. However, this approach is challenged by the possibility of crystals moving in and out of the imaging plane during long and continuous imaging, which could be misinterpreted as the crystals being selectively dissolved. Indeed, we observed that continuous imaging of MSUM crystals on a glass slide for more than 30 min resulted in a number of crystals gradually fading in and out of the image all the time. Figure 3 (upper panel) demonstrates that some crystals were moving in and out of focus plane during 2 min of observation. As small particles exhibit increased Brownian motion, we expected this problem to be more pronounced for actual MSUM crystals from gout patients, which are significantly smaller than the synthetic crystals used in this study (5.5 ± 4.5 μm^18^ vs 6.8 ± 2.5 μm, respectively). The moving crystals can be stopped by coating a glass slide with some cationic polymers such as PLL and chitosan. Therefore, the crystals were subjected to a zeta potential measurement for evaluation of the surface charge, as well as the change in the surface charge after interaction with PLL and chitosan.

**Figure 4:**
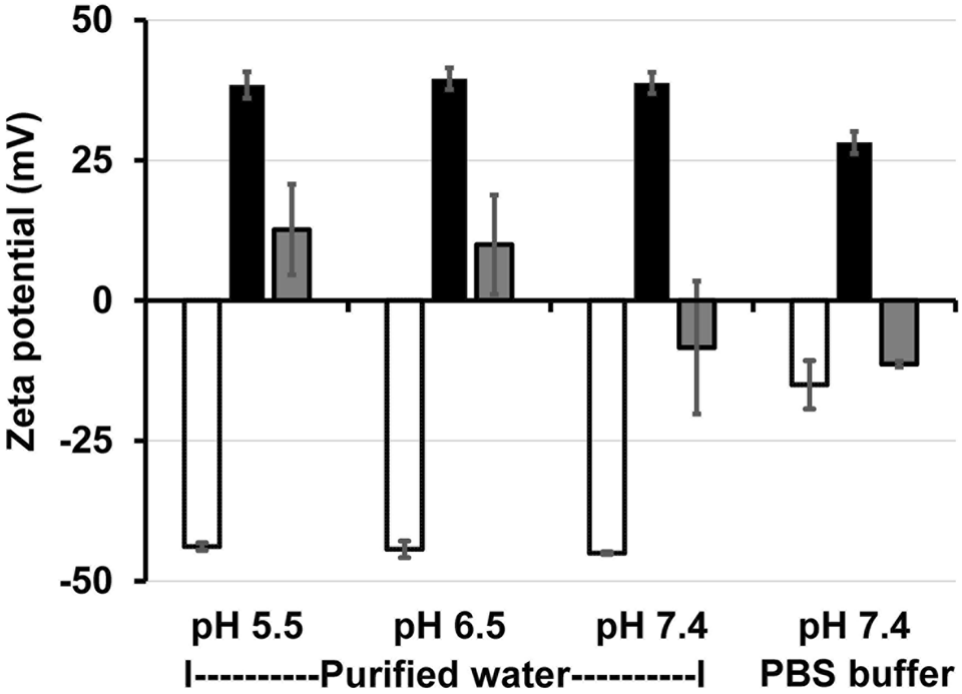
Surface property of gouty crystals. Zeta potential of MSUM crystals suspended in purified water (pH 5.5–7.4) or in 1x PBS (pH 7.4) before (white) and after mixing with 0.01% w/v PLL (black) or 0.01% w/v chitosan (gray) at the volume ratio of 9:1 (crystal suspension: polymer solution). The data represent average values and error bars represent SD (n = 3).

Zeta potential is a physical property representing an electrokinetic potential at the interface which separates mobile fluid from fluid that remains attached to the particle’s surface. Thus, it can be used to roughly estimate the surface charge of the particle. The zeta potential of MSUM in water (pH 7.4) was determined to be − 45.01 ± 0.23 mV, in good agreement with previously reported values^24^ (Fig. 4). When the crystals were mixed with 0.01% w/v PLL, however, the surface charge of MSUM turned highly positive (38.80 ± 1.88 mV), and remained so (28.18 ± 1.97 mV) even in the presence of a 1x phosphate buffer saline (PBS, 10 mM, pH 7.4) solution containing 137 mM NaCl and 2.7 mM KCl, indicating a robust attraction between PLL and MSUM (Fig. 4). The attraction was considered as electrostatic attraction because there was no chemical reaction between MSUM and PLL as confirmed by UV–visible spectrophometry (see Supplementary Fig. S4 online). In comparison, mixing chitosan and MSUM crystals in water (pH 6.5) resulted in a zeta potential of only 9.94 ± 8.87, suggesting that the attraction between MSUM and chitosan was comparatively weak (Fig. 4). Increasing the pH from 6.5 to 7.4 resulted in the solution turning turbid, presumably due to the pH being higher than the p*K*_a_ of chitosan (p*K*_a_ 6.8), which promoted the deprotonation of amino groups and consequently rendered chitosan insoluble^32^. At this pH, chitosan was no longer capable of interacting with MSUM crystals, as the addition of chitosan to MSUM in water or 1xPBS only slightly increased zeta potential from − 15.02 ± 4.31 to − 11.31 ± 0.51 mV. Similar trend was observed in the case of CPPD crystals (see Supplementary Fig. S5 online). Based on these observations, PLL was selected as the coating material for immobilizing gouty crystals.

Immobilizing MSUM crystals on a PLL-coated glass slide led to a larger number of crystals being observed with improved clarity (Fig. 3 lower panels), and similar results were obtained with CPPDs crystals. Therefore, using glass slides coated with 0.01% w/v PLL presents an effective strategy for restricting crystals motion and enhancing the accuracy of the selective dissolution method, which will be described in the subsequent sections.

### Dissolution of gouty crystals observed under microscope

**Figure 5:**
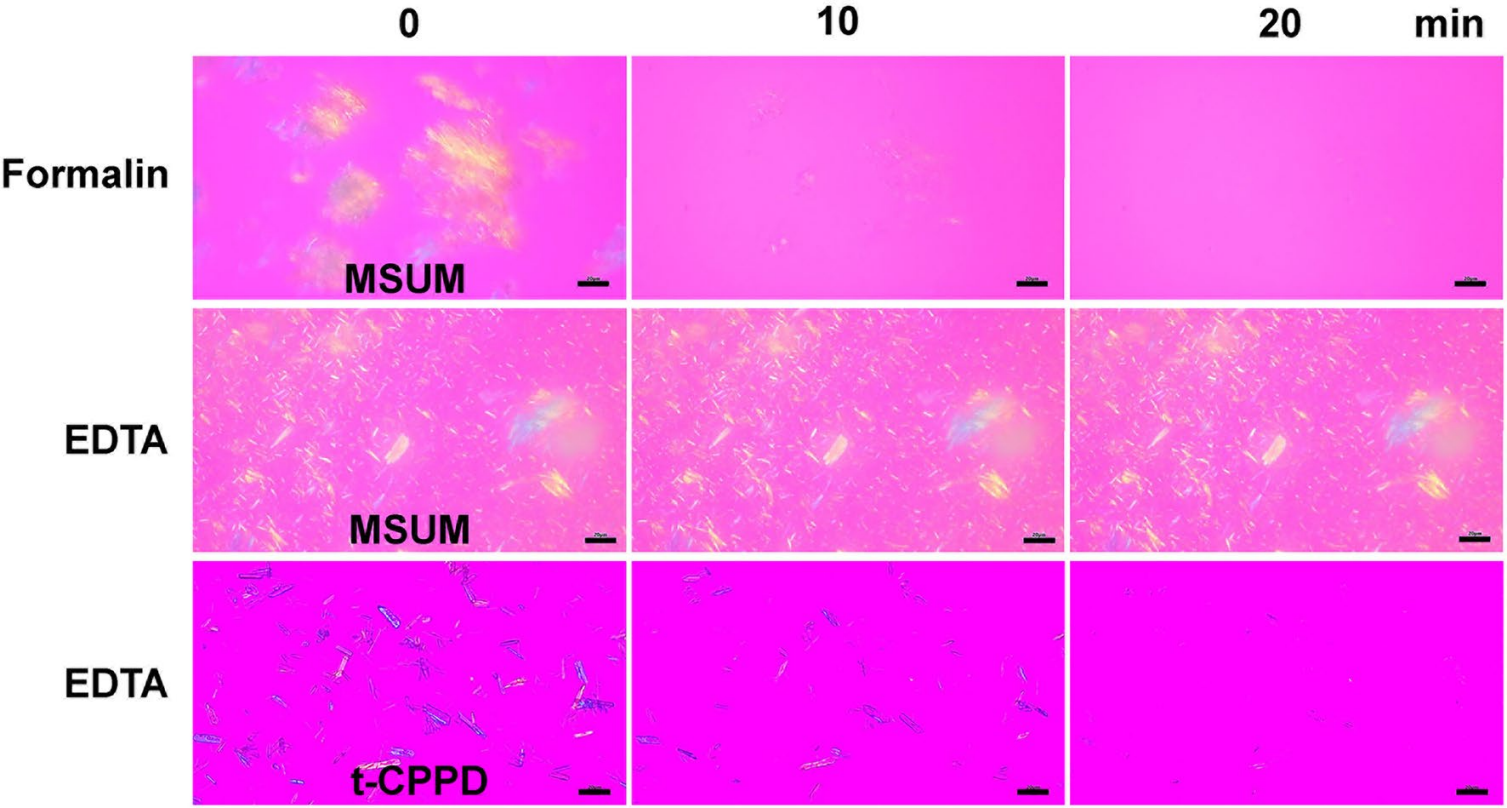
Polarized light microscopy of soaked MSUM and t-CPPD crystals on PLL-coated glass slide. MSUM crystals soaked in either (Top) 40% v/v formalin phosphate buffer (10 mM, pH 7.4) or (Middle) 10% w/v EDTA aqueous solution (pH 9.0). (Bottom) t-CPPD crystals soaked in 10% w/v EDTA aqueous solution (pH 9.0). All experiments were conducted at room temperature. Scale bars represent 20 μm. DIC images are available in Supplementary Fig. S7 online.

**Figure 6:**
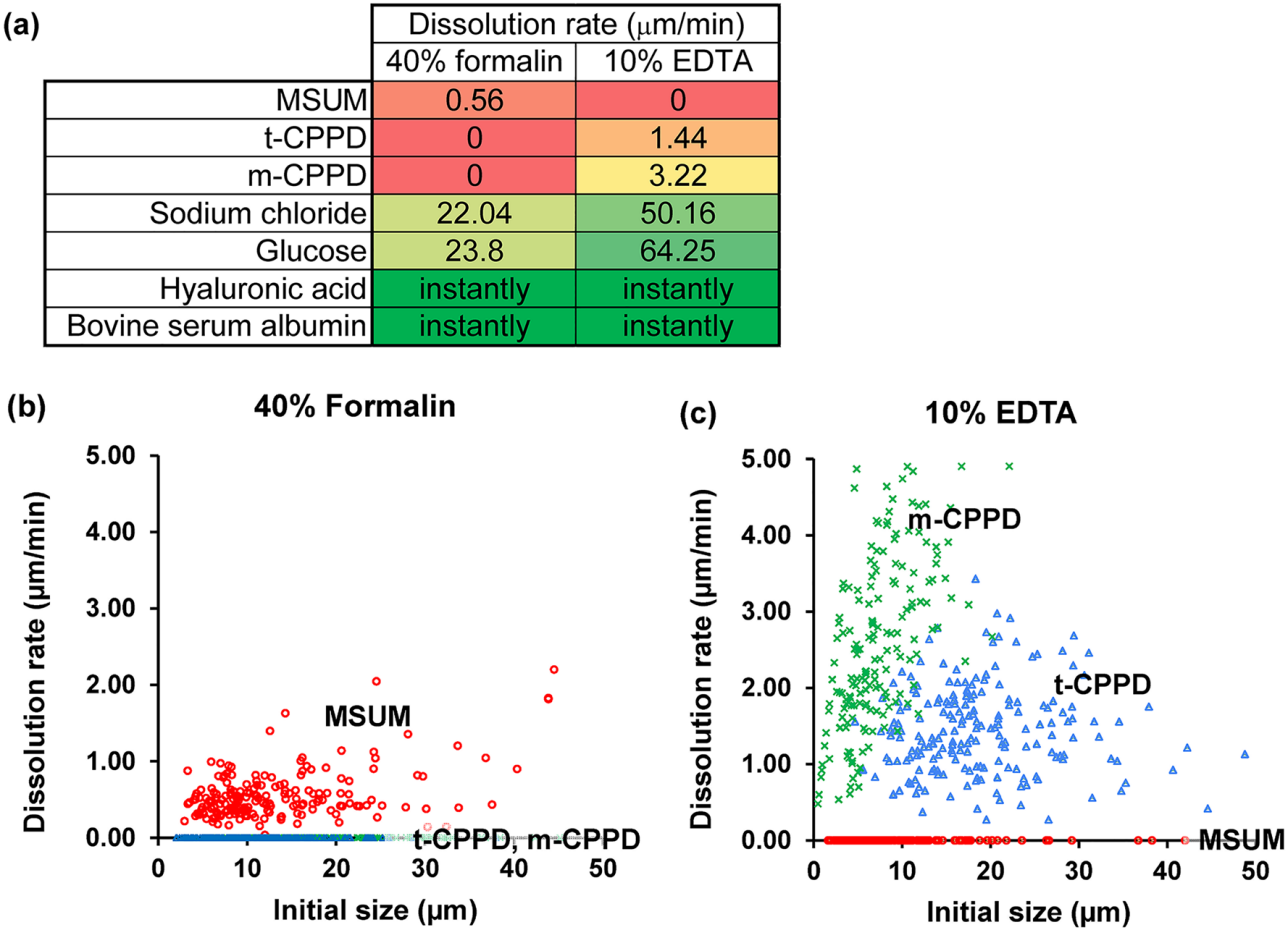
Dissolution rates of gouty crystals determined using microscopy. (**a**) Heat map showing the dissolution rates of various compounds that can be found in the synovial fluid (n = 200 for MSUM and CPPDs, n = 20 for other compounds). Red = insoluble; Yellow = moderate soluble; Green = very soluble. Scatter plots between initial size of crystals and dissolution rate in (**b**) 40% v/v formalin phosphate buffer (10 mM, pH 7.4) and **(c)** 10% w/v EDTA aqueous solution (pH 9.0) (n = 200). Red circle: MSUM; blue triangle: t-CPPD; green cross: m-CPPD.

Before examination under a polarized light microscope, MSUM crystals were soaked in 1–2 μL of 40% v/v formalin phosphate buffer (10 mM, pH 7.4) and placed on a PLL-coated glass slide. Almost all crystals disappeared within 20 min of contact (Fig. 5). In contrast, soaking the crystals in 10% w/v EDTA aqueous solution (pH 9.0) did not affect the number and shape of crystals, confirming that the MSUM crystals did not dissolve in the EDTA solution (Fig. 5). To calculate the dissolution rate of each type of crystals, the length of crystals over time were tracked using microscopy. The results show that, in 40% formalin phosphate buffer (pH 7.4), the average dissolution rate of MSUM crystals (n = 200) was 0.56 ± 0.33 μm/min (Fig. 6a). Conversely, the dissolution rate of MSUM in 10% w/v EDTA aqueous solution (pH 9.0) was 0, in line with the results of the gravimetric analysis. The experiment was repeated with t-CPPD and m-CPPD as shown in Fig. 6b,c, and other relevant compounds that may be present in the synovial fluid or biological milieu, such as sodium chloride, glucose, the solid form of hyaluronic acid and bovine serum albumin (BSA). Hyaluronic acid and BSA dissolved instantly in both 40% formalin and 10% EDTA, while sodium chloride and glucose dissolved more rapidly in 10% EDTA than 40% formalin (Fig. 6a). An average dissolution rate of m-CPPD crystals in 10% w/v EDTA aqueous solution (pH 9.0) was highest (3.22 ± 2.17 μm/min), followed by t-CPPD (1.44 ± 0.58 μm/min) (Fig. 6a). Therefore, 10% w/v EDTA aqueous solution (pH 9.0) is employed further to eliminating the possible interference for MSUM detection under microscope. In contrary, the use of formalin solution for interferences elimination is also highly possible for the detection of CPPDs. However, polarized light microscope cannot be applied for the m-CPPD detection because single crystals of m-CPPD displays almost no birefringence and can be observed under bright field only, while the aggregated m-CPPD displayed some weak birefringence (see Supplementary Fig. S6 online).

While 40% formalin appeared to be effective in dissolving MSUM crystals, there remained a concern of potential reactivity between uric acid and the components of the solvent system. Despite a previous report that boiled uric acid could react with formalin to produce a resinous solid^33^, no such solid was observed after MSUM was soaked in 40% formalin for 1 h in this study. To further confirm the integrity of the uric acid molecule as a primary component of MSUM, the soaked solution was subjected to UV–visible spectroscopy and electrospray ionization mass spectrometry (ESI–MS). The UV–visible spectra of dissolution product displayed a similar profile to that of uric acid and remained unchanged throughout 60 min of observation. Moreover, the negative mode mass spectra acquired at 0, 30 and 60 min showed peaks at m/z of 167 and 124, corresponding to uric acid [M–H]^−^ and its fragment [M–H–NHCO]^−^, respectively. The ESI–MS result was in good agreement with the previous report^34^. No other peaks indicative of uric acid degradation were observed. These results confirmed that the 40% formalin system is effective in dissolving MSUM crystals and did not cause undesirable chemical reactions (see Supplementary Figs. S8 and S9 online).

### Detection of gouty crystals in spiked porcine synovial fluid

**Figure 7:**
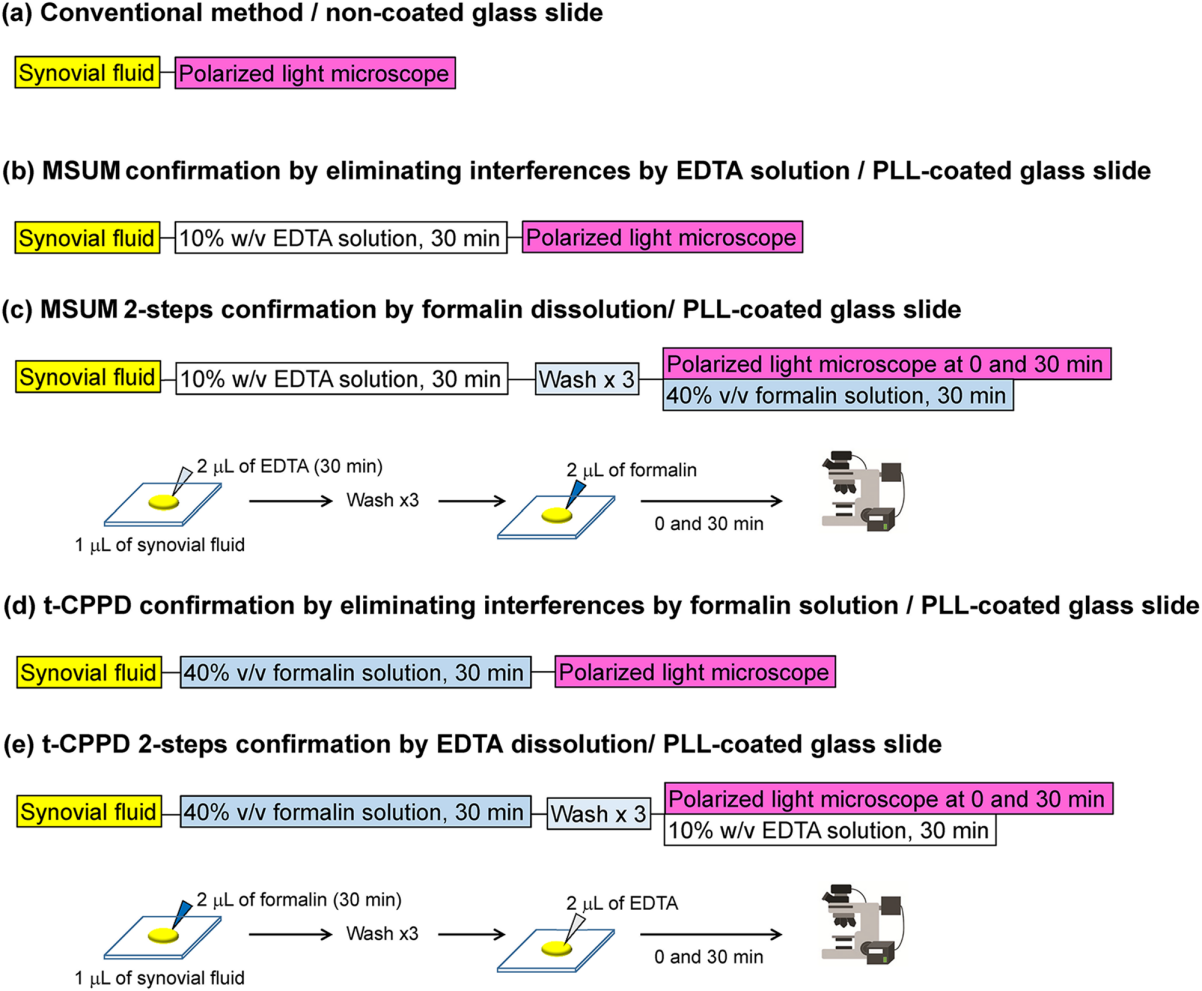
Diagrams summarizing methods for gouty crystals detection in synovial fluid. (**a**) Conventional crystal identification using birefringence. (**b**) Elimination of interferences via dissolution in 10% w/v EDTA aqueous solution (pH 9.0) for observation of MSUM birefringence. (**c**) MSUM 2-steps confirmation by EDTA following by dissolution in 40% v/v formalin phosphate buffer solution (pH 7.4). (**d**) Elimination of interferences via dissolution in 40% v/v formalin phosphate buffer solution (pH 7.4) for observation of t-CPPD birefringence. (**e**) t-CPPD 2-steps confirmation by formalin following by dissolution in 10% w/v EDTA aqueous solution (pH 9.0).

**Figure 8:**
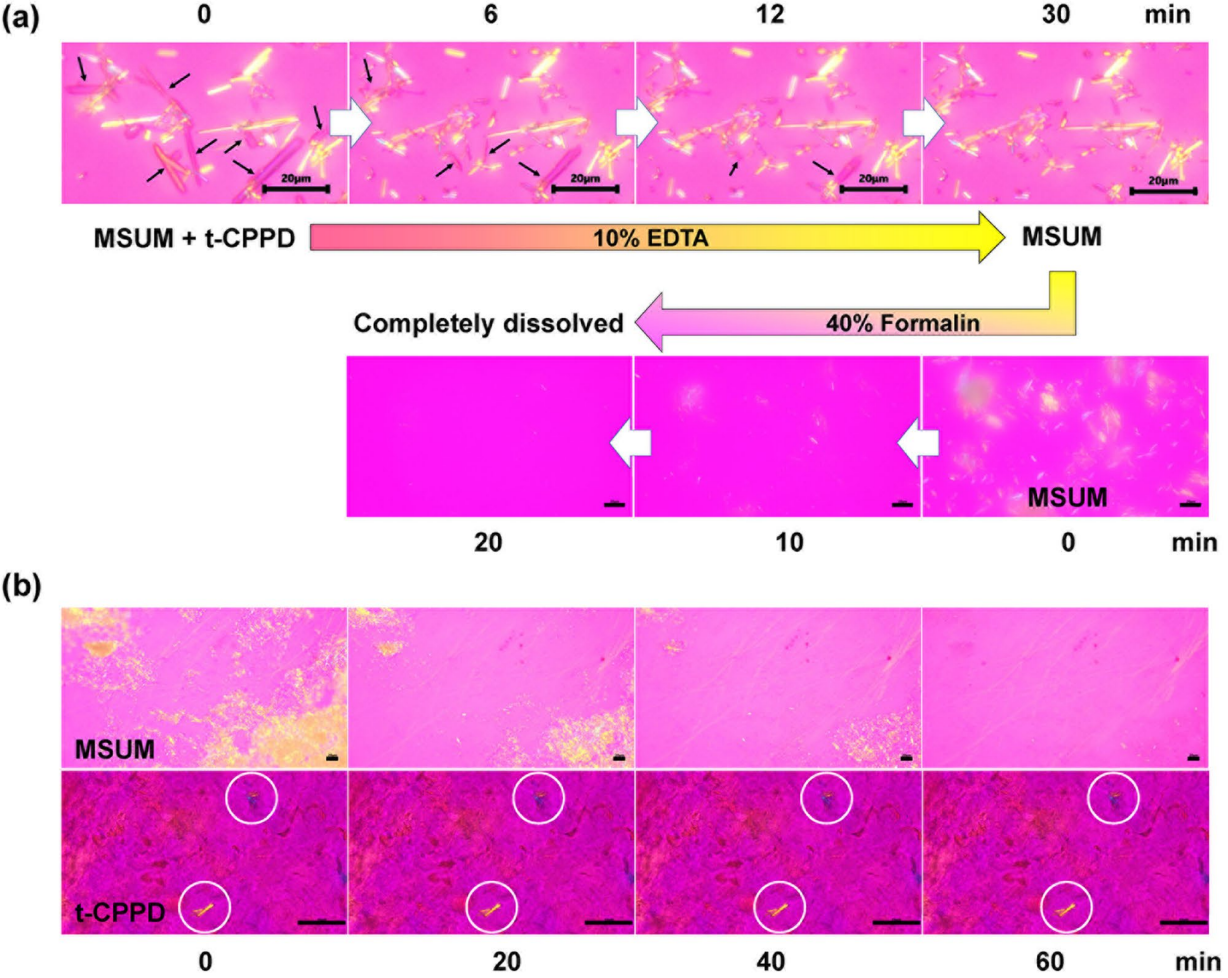
Gouty crystals detection in biological specimen. (**a**) Two-steps confirmation of MSUM in t-CPPD mixed sample spiked in porcine synovial fluid (1 μL) after addition with 10% w/v EDTA solution (pH 9.4, 2 μL). The droplet was washed, blotted and then added 40% v/v formalin phosphate buffer solution (10 mM, pH 7.4, 2 μL) on PLL-coated glass slide as illustrated in Fig. 7c. Black arrows indicate the t-CPPD crystals. (**b**) Dissolution of MSUM (yellow birefringence) and persistence of t-CPPD (yellow and blue birefringence shown in white circles) in rat tissue specimens placed on a non-coated glass slide under a polarized light microscope soaked with 40% v/v formalin phosphate buffer (10 mM, pH 7.4). Scale bars represent 20 μm for MSUM and 50 μm for t-CPPD. DIC and bright field images are available in Supplementary Fig. S10 online.

Detection of crystals in synovial fluid samples has been regards as a gold standard for gouty diagnosis^35^. However, relying solely on birefringence to distinguish between MSUM and CPPD has been challenging due to the color varying with the angle of the polarized light (Fig. 5). Furthermore, although MSUM displays stronger birefringence than t-CPPD, the distinction may not be apparent to untrained eyes, leading to high false negative rates (2.8% for gout patients and 7.9% for pseudogout patients)^9^. This problem of accuracy is further exacerbated when both MSUM and CPPD crystals are present in a patient’s synovial fluid^3,36^. To enhance the sensitivity of a conventional polarized light microscopy for the detection of MSUM in synovial fluids (Fig. 7a), we investigated the use of 10% w/v EDTA aqueous solution to remove CPPDs and other interferences (Fig. 7b). A spiked porcine synovial fluid (1 μL) was incubated with 10% EDTA (2 μL, pH 9.0) for 30 min on a PLL-coated glass slide, after which it was inspected using polarized microscopy. The results show that all of the t-CPPD crystals disappeared within 30 min (Fig. 8a). The crystals can then be washed with purified water and dissolved in 40% formalin for the 2-steps confirmation of MSUM crystals (Figs. 7c and 8a). The confirmation of t-CPPD in the synovial fluid was also performed in one or two steps as shown in Fig. 7d,e.

### Detection of gouty crystals in rat tissues

To assess the generalizability of our proposed methods, they were additionally tested using the rat subcutaneous air pouch model. Crystals were injected into a rat air pouch and, after 24 h, tissues containing white MSUM aggregates or individual CPPDs were collected and cryo-processed on a dry ice cube. The tissues were then cryo-sectioned at 10 µm thickness and soaked in 40% v/v formalin phosphate buffer on a non-coated glass slide. The crystals-induced inflammatory response in the air pouch was confirmed by counting white blood cells in the exudate (see Supplementary Fig. S11 online).

Interestingly, at 24 h after each crystal injection, MSUM crystals showed a strong tendency to aggregate and form tophi in the inflamed rat air-pouch tissue. Under a polarized light field, clumpy MSUM crystals or tophi were clearly visible with yellow birefringence (Fig. 8b). Incubating the tissue with 40% formalin buffer (pH 7.4) resulted in the complete disappearance of the crystals after 60 min as clearly shown in Fig. 8b. In comparison, the t-CPPD crystals remained no change after 60 min of incubation. Therefore, dissolution with formalin could assist the confirmation of MSUM crystals in the rat tissue observed by polarized light microscope. The confirmation of t-CPPD crystals in the rat tissue with 10% w/v EDTA solution can also be performed in similar manner.

## Conclusions

In summary, we have reported a simple method for improving the sensitivity and specificity of gouty crystal detection using polarized light microscopy. The 40% formalin buffer solution was found to selectively dissolve MSUM while the 10% EDTA solution selectively dissolved CPPDs, offering a promising strategy for reducing false positives. The strategies reported herein were also applicable in complex biological milieu, including spiked porcine synovial fluid and rat air-pouch tissue. Further detection of m-CPPD crystals by microscopic method is under investigation.

## Methods

### Chemicals and materials

All chemical reagents were of analytical grade and used without further purification. Chitosan (Product No. 448869, deacetylation degree of 91.7%, MW of 359 kDa) and 0.1% (w/v) poly-*L*-lysine (PLL) solution were purchased from Merck KGaA, (Darmstadt, Germany). Formaldehyde solution (36–38%) was commercially available from Kemaus (New South Wales, Australia). Uric acid was purchased from Fujifilm Wako chemical (Tokyo, Japan). All aqueous solutions were prepared using deionized water from a Milli-Q water purification system (Millipore, Bedford, MA, USA). Tissue-Tek^®^ O.C.T. compound was commercially available from Sakura FineTek (USA). Porcine synovial fluids were agricultural waste obtained from the hind legs of 90–120 kg adult healthy pigs from a local slaughter house (Betagro Public Company, Banglen, Nakhon Pathom, Thailand). Synovial fluid was removed from the knee joint, located 22 cm away from the end of a leg. This procedure was performed using a 20-gauge needle, approximately 1 h after death. The samples were stored at − 20 °C until use. The protocol of porcine synovial fluid collection was approved by the Faculty of Science, Mahidol University Animal Care and Use Committee (Ethical clearance Protocol No. MUSC66-013-643).

### Synthesis and characterization of MSUM crystals

The synthesis of small MSUM crystals was modified from the protocol described by Denko and Whitehouse (1976)^37^. A total of 2.00 g of uric acid was weighed and dissolved in 400 mL of deionized water under ambient air. The solution was then continuously stirred and heated at 60 ℃ for 30 min. Subsequently, the pH of the solution was adjusted to 8.9 using 2 M NaOH, resulting in a clear solution. The solution was then cooled to room temperature and subjected to vigorous stirring at 900 rpm for 12 h. The resulting white powder was filtered through a Whatman grade 1 filter paper and dried in an oven at 30 ℃ for 24 h. The final yield of MSUM crystals was 2.395 g (96.13% yield). The crystals were stored in a sealed glass vial inside a desiccator at room temperature until further use. X-ray diffraction measurements were performed with a Bruker AXS model D8 discover equipped with Cu radiation (40 mA current and 40 kV voltage). The crystals were scanned over the 2θ range of 2–70°, with a scanning rate of 0.0116° per minute at 25 °C. FT-IR spectra of the crystals were acquired using a PerkinElmer frontier FTIR with a resolution of 4 cm^−1^ and 16 scans per spectrum in the wavenumber range of 4000–400 cm^−1^. Raman spectroscopy was performed to characterize the crystal using a Horiba XploRA PLUS confocal Raman microscope over the wavenumber range of 50–3400 cm^−1^, with the wavelength of the laser source set at 532 nm. Thermal gravimetric analysis was performed with a PerkinElmer TGA 4000 over the temperature range of 30–500 °C at a heating rate of 5 °C per min.

### Gravimetric analysis of gouty crystals dissolution

Totally, 7 samples of MSUM crystals (50 mg) were weighed and dissolved in 5 mL of either 10–40% v/v formalin phosphate buffer solution (10 mM, pH 7.4) or 10–30% w/v EDTA aqueous solution (pH 9.0). All samples were places in a shaking incubator at 25 °C at a speed of 200 rpm. At time interval, the samples were filtered by vacuum filtration with filter paper (Whatman^®^ Grade 1 with 11-µm pore size) and washed with distilled water (2 mL) to obtain the remaining crystals. The samples were dried in a hot-air oven at 180 °C for 2 h and then placed in a desiccator overnight. The weights (mg) of filter paper before and after filtration including remained solid were recorded. The drying process was repeated until the sample was completely dried and a constant weight was obtained. The difference in weights of filter paper before and after filtration obtained from each sample indicated the remaining weight of crystals on the filter paper at each condition. Similar procedure was conducted in the case of t-CPPD and m-CPPD.

### Interaction of MSUM crystals with PLL

The obtained gouty crystals were resuspended in a pH-adjusted water that had been sterilized via a 0.45 µm syringe filter. A 0.01% w/v PLL solution was prepared by diluting a commercial 0.1% w/v PLL solution (Sigma-Aldrich, USA) in purified water. A stock of 1% w/v chitosan in 1% acetic acid was diluted 100-fold with purified water to obtain a 0.01% w/v chitosan solution. Crystal suspensions (1 mg/mL) with a pH of 5.5, 6.5 and 7.4 were mixed with either 0.01% w/v PLL solution or 0.01% w/v chitosan in 9:1 v/v ratio. The pH of the mixtures was re-adjusted to 5.5, 6.5 and 7.4. The zeta potential of the samples was analyzed using a Zetasizer Nano ZS (Malvern Instrument, UK). The interaction between crystals and cationic polymers in phosphate buffer saline (PBS, pH 7.4) was also assessed to elucidate the effect of ionic strength on the attachment of polymers to the crystals. All experiments were performed in triplicates.

### Microscopic observation of gouty crystals dissolution

A concave glass slide was treated with 34% nitric acid overnight, rinsed with double distilled water twice, and then rinsed with HPLC grade methanol. The pretreated glass slide was immersed in the HPLC grade ethanol until use. A glass coverslip was coated with 0.01% w/v PLL by soaking it in a 0.01% w/v PLL solution for 10 min and then washed 3 times with double distilled water. The coverslip was dried under clean air for at least 2 h before use. To prepare a sample for microscopic observation, synthetic gouty crystals were added to a 2 μL drop of 40% v/v formalin phosphate buffer solution (10 mM, pH 7.4) placed on the coated coverslip. Then, the specimen was subjected to the hanging drop technique, with the coverslip mounted on a concave glass slide, facing downward. The morphology and birefringence of the stained crystals were observed under a fluorescence microscope (Olympus BX51, Japan) equipped with a U-GAN gout analyzer. The dissolution rate of each type of crystals was determined by measuring the change in crystal length (μm) per min.

### Animal experiment

Male Wistar rats (7–8 weeks, 200–220 g) were housed in pairs and allowed to acclimatize to the new environment for at least 5–7 days before randomly assigned to three groups. To induce the inflammation of their subcutaneous air pouch, anesthesia was induced using 5% isoflurane and maintained with 3% isoflurane inhalation as previously described^38^. Deep and stable anesthesia was confirmed by a toe pinch at the webbing of a hind paw. Then, the dorsal area of the rats was shaved and cleaned with povidone iodine, and 20 mL of sterile air was injected subcutaneously. Air pouches were reinjected with 10 ml of sterile air on the third day to maintain inflation. Six days after the air pouch was formed, rats in Group 1 (n = 6) were injected with the vehicle (HEPES buffer saline (HBS), 10 mL), while Group 2 and 3 (n = 6 each) were injected with synthetic MSUM (15 mg in 10 mL HBS) and t-CPPD crystals (15 mg in 10 mL HBS), respectively. At 24 h post crystal injection, animals were humanely euthanized with an overdose of thiopental (100 mg/kg; i.p.) and the exudate of each air pouch fluid was collected to determine the white blood cells count. The air pouch tissue was dissected and cryo-embedded, followed by microscopic observation of the crystals. All animal experiments were approved by the Faculty of Science, Mahidol University Animal Care and Use Committee (Bangkok, Thailand) with approval number MUSC63-040-548. All methods were performed in accordance with the relevant guidelines and regulations. This study is reported in accordance with ARRIVE guidelines (Animal Research: Reporting of in vivo Experiments; https://arriveguidelines.org).

### Detection of gouty crystals in spiked porcine synovial fluid and rat air-pouch tissue

The porcine synovial fluid was spiked with synthetic gouty crystals (approximately 0.04 mg/μL) using a vortex mixer. A 1-μL drop of the spiked fluid was gently placed on a PLL-coated coverslip, and 40% v/v formalin phosphate buffer solution (10 mM, pH 7.4, 2 μL) was added to the droplet. The specimen was then subjected to the hanging drop technique. The morphology and birefringence of the stained crystals were observed using a fluorescence microscope (Olympus BX51, Japan) equipped with a U-GAN gout analyzer. For the rat air-pouch tissue, an appropriate volume of 40% v/v formalin phosphate buffer solution (10 mM, pH 7.4) was added to cover the 10-µm-thickness cryo-sectioned specimen, and imaging was performed with the glass slide facing upward. Addition of an appropriate volume of 40% v/v formalin phosphate buffer solution (10 mM, pH 7.4) to the specimen at 10 min time interval was needed during observation.

### Statistical analysis

Statistical analysis was performed using the PASW Statistics SPSS software (v18.0, SPSS, Inc., Chicago, IL, USA). Distribution of data was tested using the Shapiro–Wilk test and homogeneity of variances was assessed using Levene’s test. Data was log-transformed prior to perform statistically significant. Data was analyzed with one-way ANOVA, in which the Bonferroni post hoc test was used to compare multiple groups. All data are presented as the mean ± standard deviation (SD) unless otherwise stated. *P* values of ≤ 0.05 were regarded as significant.

## Supplementary Information

Supplementary Information.

## Data Availability

All data generated or analyzed during this study are included in this published article and its Supplementary Information file.
