# Supplementary material for: Improved polarized light microscopic detection of gouty crystals via dissolution with formalin and ethylenediamine tetraacetic acid
Source: Sci Rep. 2023 May 9;13:7505. doi: 10.1038/s41598-023-34570-5 (PMC10170089; doi:10.1038/s41598-023-34570-5)
Supplement: Supplementary file 1 — Supplementary Information. [file 41598_2023_34570_MOESM1_ESM.pdf]

Supplementary information

Improved Polarized Light Microscopic Detection of Gouty Crystals via Dissolution with Formalin and Ethylenediamine Tetraacetic Acid

Ruedee Hemstapat[1], Peeradon Duangiad[2], Borwornporn Tangketsarawan[1,2], Thitiya Phuagpan[2], Sinthida Chienwiwattanawong[2], Nuttinee Tangsrianugul[2], Akio Ojida[3], Jirarut Wongkongkatep[2]*

[1]Department of Pharmacology, Faculty of Science, Mahidol University, 272 Rama 6 Road, Bangkok 10400 Thailand

[2]Department of Biotechnology, Faculty of Science, Mahidol University, 272 Rama 6 Road, Bangkok 10400 Thailand

[3]Graduate School of Pharmaceutical Sciences, Kyushu University, 3-1-1 Maidashi, Higashi-ku, Fukuoka 812-8582 Japan

Synthesis and characterization of CPPD crystals

The methods for monoclinic (m-) and triclinic (t-) CPPD synthesis and characterization were followed Srinarawat et al. (2022). The ammonium acetate buffer was prepared by mixing 6 ml of glacial acetic acid with 200 ml of deionized water, before the pH was adjusted with 25% ammonia to 5.8 and 3.6 for the synthesis of m- and t-CPPD, respectively. The buffer was continuously stirred and heated until the temperature reached 90°C. Next, 100 ml of CaCl2 aqueous solution (76.25 mM for m-CPPD and 302.97 mM for t-CPPD synthesis) and 100 ml of sodium pyrophosphate decahydrate (NaPPi, 50.35 mM for m-CPPD and 151.02 mM for t-CPPD synthesis) were fed simultaneously into the buffer solution at feeding rate of 4.5 ml/min using Masterflex L/S® 77202-50 (Masterflex, USA). After 22 min of vigorous stirring, the feeding was stopped and the solution was heated for 1 h. The solution was filtered and the residue was dried in a hot air oven at 180°C for 2 h. The dried weights of the obtained m- and t-CPPD crystals were 1.46 g (97.6% yield) and 4.11 g (93.8% yield), respectively. The average size of m-CPPD was 17.1±3.3 µm in length while the size of t-CPPD were 13.7±3.8 micrometer long and 2.7±0.7 micrometer wide (n=50). The crystals were kept in a sealed glass vial and stored inside a desiccator at room temperature. XRD measurements were performed with a Bruker AXS model D8 discover equipped with Cu radiation set at 40 mA current and 40 kV voltage. The crystals were scanned from 2-70° in the 2θ range with a scanning rate of 0.0116° per minute at 25°C. FT-IR spectrums of the crystals were characterized by a PerkinElmer frontier FTIR with 4 cm^{-1} resolutions and 16 scans per spectrum over the wavenumber range of 4000 - 400 cm^{-1}. Raman spectroscopy was performed to characterize the crystal using a Horiba XploRA PLUS confocal Raman microscope over the wavenumber range of 50-3400 cm^{-1} with laser source at 532 nm. TGA was performed with a PerkinElmer TGA 4000 in the temperature range of 30-500°C at a heating rate of 5°C per min.

Reference:

Srinarawat, W. et al. Fluorescence identification of arthropathic calcium pyrophosphate single crystals using alizarin red S and a xanthene dipicolylamine Zn^{II} complex. *Analyst* **147**, 4910-4918 (2022).

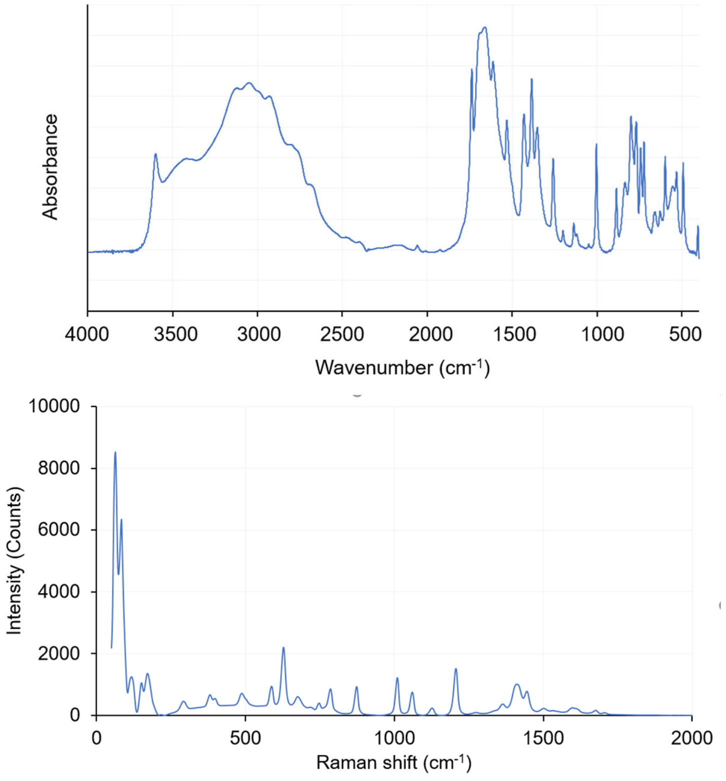

Figure S1. FTIR (PerkinElmer frontier FTIR, above) and Raman spectra (Horiba XploRA PLUS confocal Raman microscope, below) of monosodium urate monohydrate (MSUM) crystals synthesized in this study.

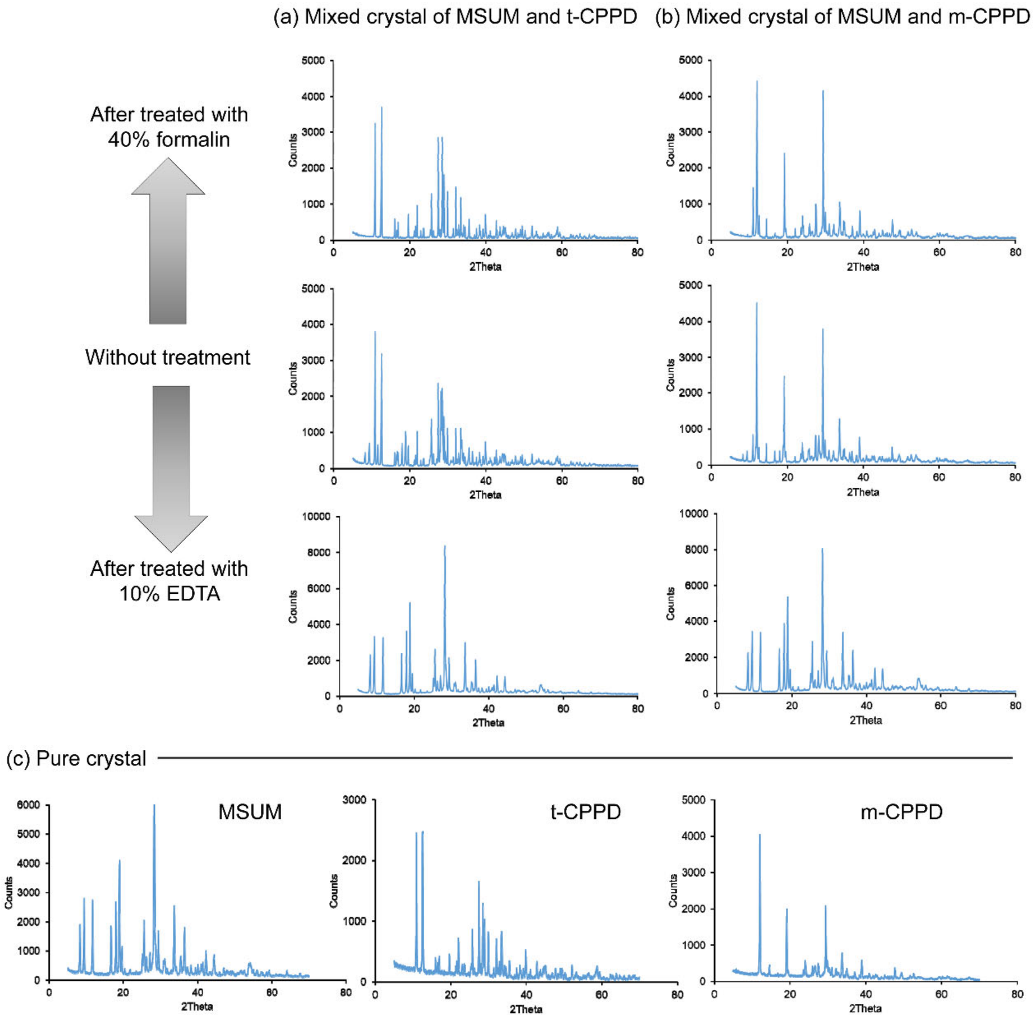

Figure S2. Powder XRD patterns (Bruker AXS D8) of the mixed crystal (1:1 by weight) between (a) MSUM and t-CPPD (b) MSUM and m-CPPD before and after soaked in 40% formalin phosphate buffer solution (pH 7.4) or 10% w/v EDTA solution (pH 9.0) for 30 min. (c) Powder XRD patterns of pure crystal for comparison.

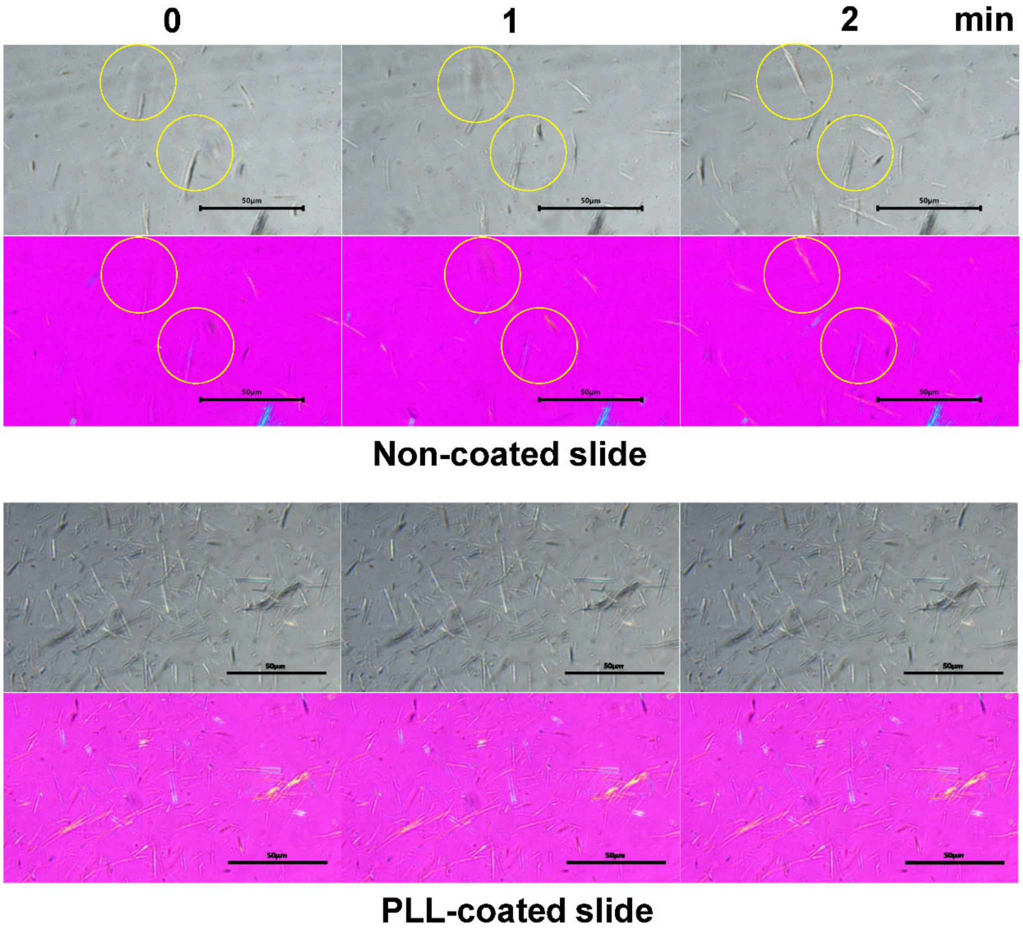

Figure S3. DIC (gray background) and polarized light (pink background) images of comparing the appearance of MSUM crystals on PLL-coated and non-coated glass slides.

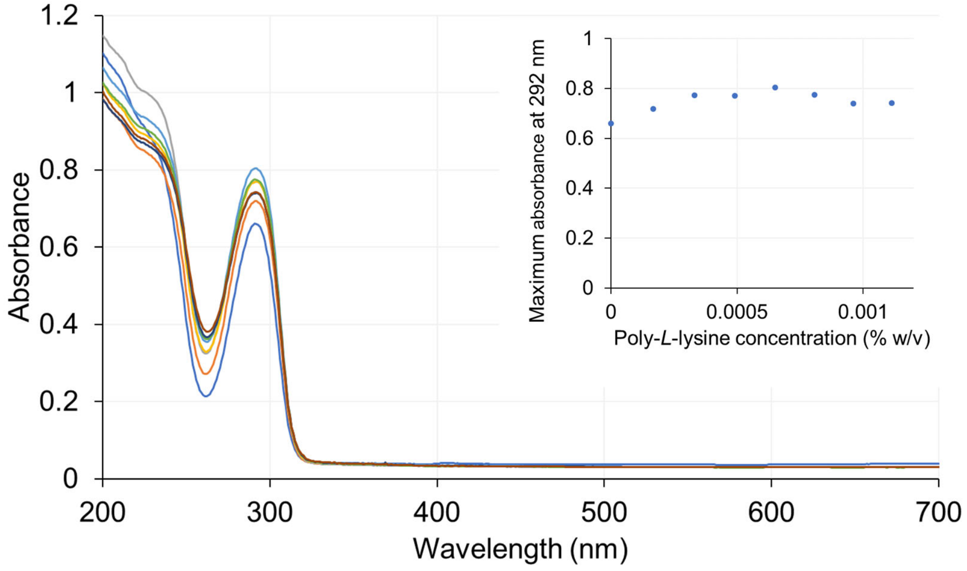

Figure S4. UV-visible spectra (JASCO V730) of uric acid (0.1 mM) as a major composition of MSUM when poly-*L*-lysine (PLL) was added at different concentration during 60 min of observation at room temperature.

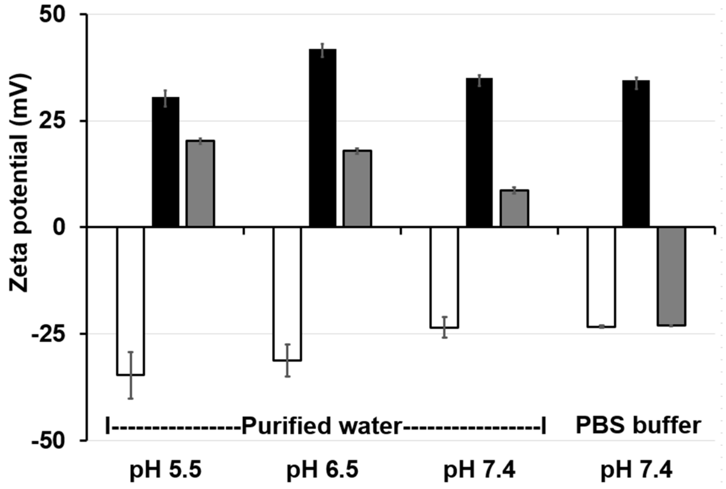

Figure S5. Zeta potential of m-CPPD crystals suspended in purified water (pH 5.5-7.4) or in 1x PBS (pH 7.4) before (white) and after mixing with 0.01% w/v PLL (black) or 0.01% w/v chitosan (gray) at the volume ratio of 9:1 (crystal suspension: polymer solution). The data represent average values and error bars represent SD (n=3).

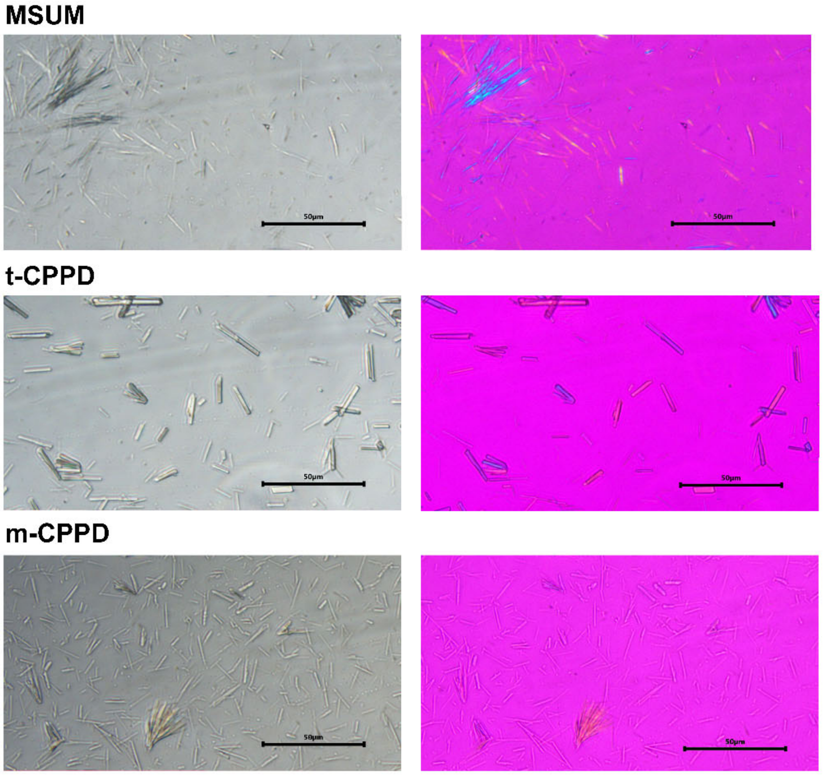

Figure S6. DIC (left panel) and polarized light (right panel) images of MSUM, t-CPPD and m-CPPD observed under a fluorescence microscope (Olympus BX51, Japan) equipped with a U-GAN gout analyzer. Scale bars represent 50 μm.

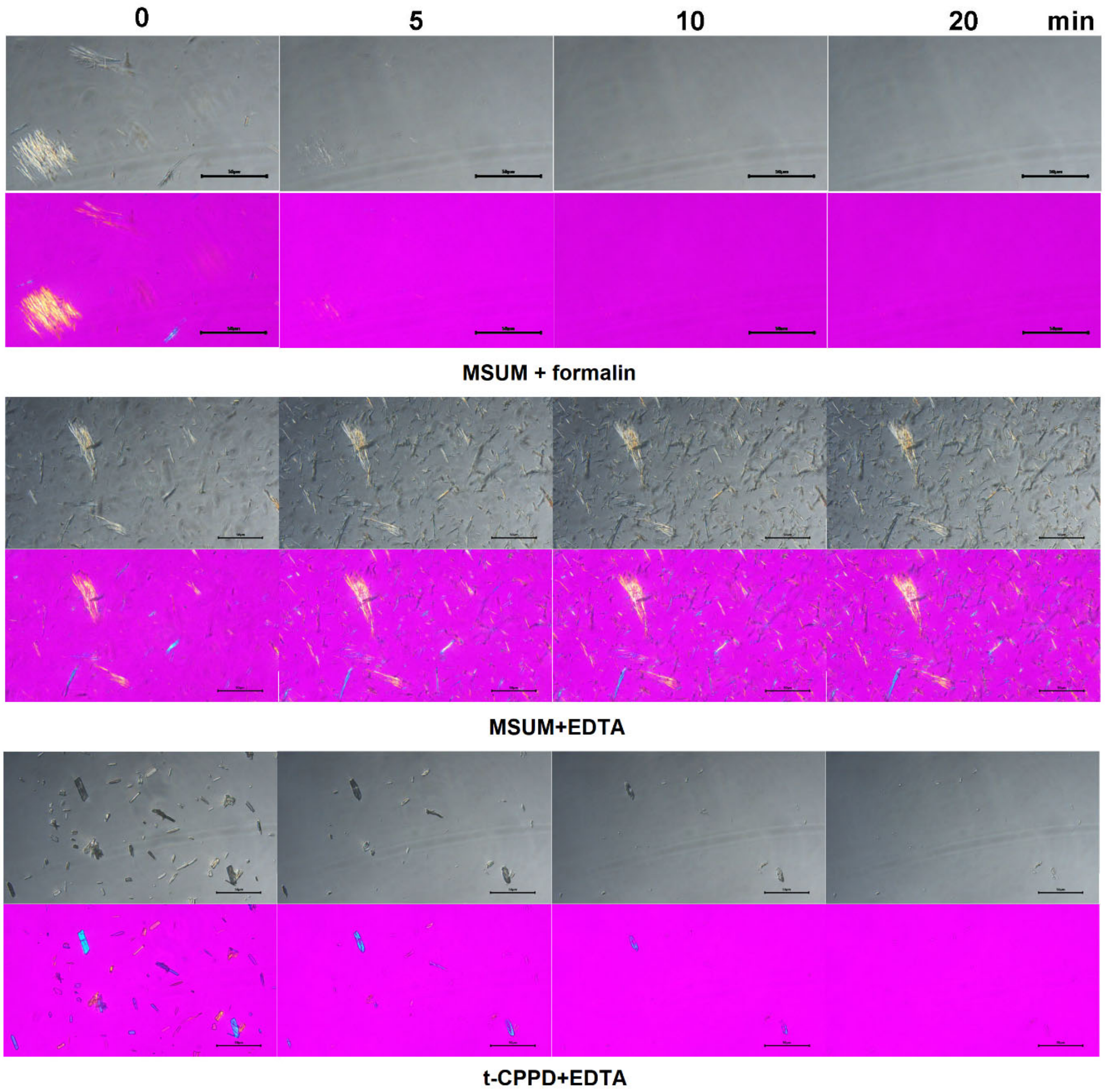

Figure S7. DIC (gray background) and polarized light (pink background) images of soaked MSUM and t-CPPD crystals on PLL-coated glass slide. MSUM crystals soaked in either (Top) 40% v/v formalin phosphate buffer (10 mM, pH 7.4) or (Middle) 10% w/v EDTA aqueous solution (pH 9.0). (Bottom) t-CPPD crystals soaked in 10% w/v EDTA aqueous solution (pH 9.0). All experiments were conducted at room temperature. Scale bars represent 50 μm.

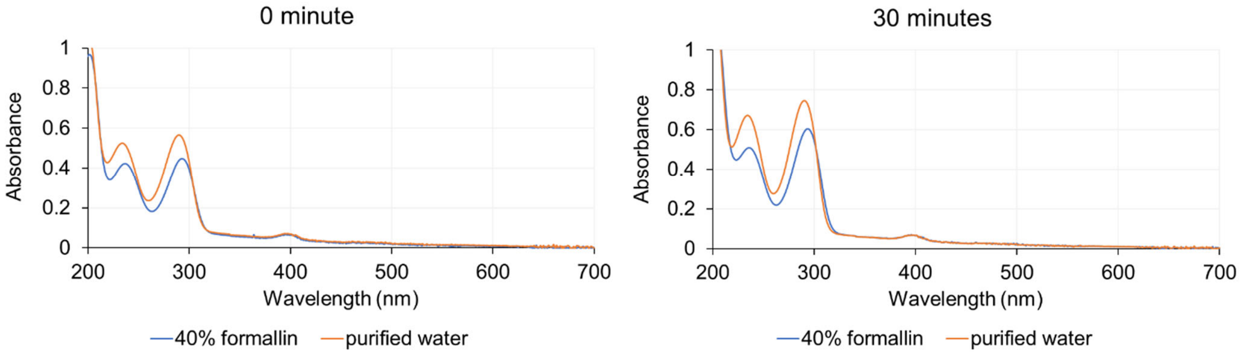

Figure S8. UV-visible spectra (JASCO V730) of supernatant after centrifugation at 9677g for 1 min of the MSUM crystals soaked in 40% formalin phosphate buffer solution (pH 7.4) or purified water (pH 7.4) for 0 and 30 min. The proper dilution of the sample was performed as needed.

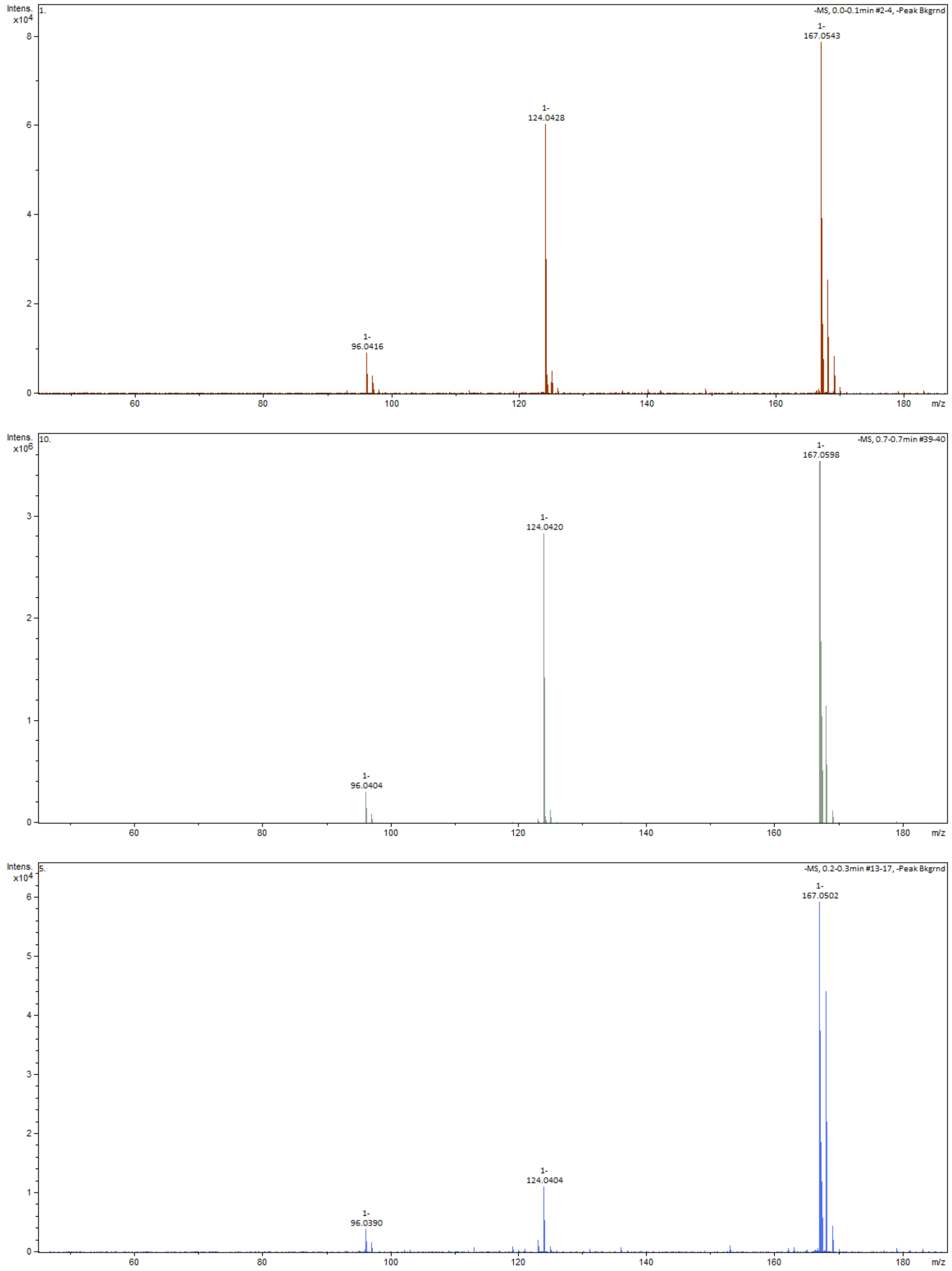

Figure S9. Mass spectra (Bruker maXis™ Ultra High Resolution time of flight Mass Spectrometer) of the 40% formalin phosphate buffer solution (10 mM, pH 7.4, 1 mL) containing 1 mg MSUM crystals at different contact time of 0 (above), 30 (middle) and 60 min (below).

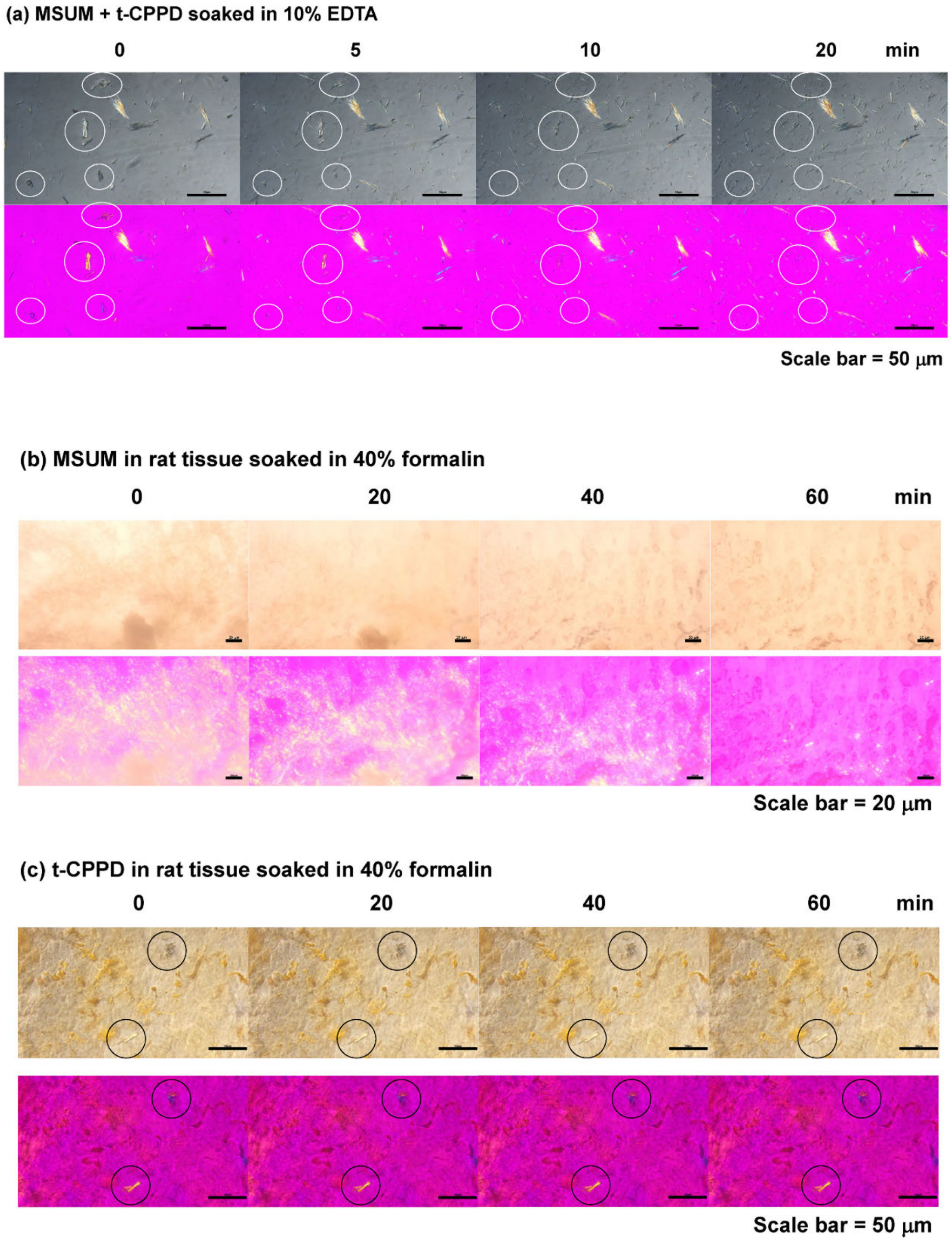

Figure S10. (a) MSUM and t-CPPD mixed crystals soaked in 10% w/v EDTA (pH 9.0) for 20 min. White circles indicate the t-CPPD crystals and their position after dissolved. (b) Dissolution of MSUM (yellow birefringence) and (c) persistence of t-CPPD (yellow and blue birefringence shown in black circles) in rat tissue specimens (10 μm-thickness) placed on a non-coated glass slide under a bright field and polarized light microscope soaked with 40% v/v formalin phosphate buffer (10 mM, pH 7.4).

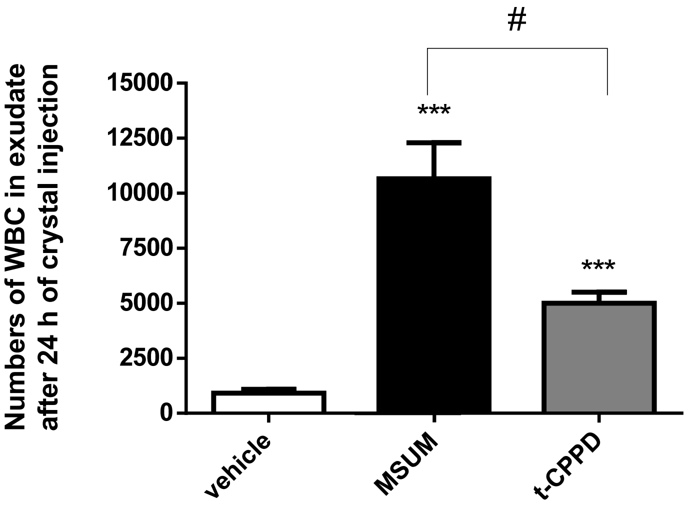

Figure S11. Numbers of white blood cells (cells/mm^3) in the exudate collected after 24 hours of crystal injection obtained from air pouch model. All data are presented the mean ± SEM of 6 rats per group. (*, *** indicate $p < 0.05$, 0.001 compared with vehicle group, # indicates $p < 0.05$. Statistical analysis was performed using one-way ANOVA followed by Bonferroni's multiple comparisons test.
